# COVID-19 Therapeutics: Improvise—Adapt—Learn

**DOI:** 10.3390/jcm11185312

**Published:** 2022-09-09

**Authors:** Joseph Abraham, Leonidas Palaiodimos, Shitij Arora

**Affiliations:** 1 Albert Einstein College of Medicine, New York, NY 10461, USA; 2 Department of Medicine, Division of Hospital Medicine, Jacobi Medical Center, NYC Health + Hospitals, New York, NY 10461, USA; 3 School of Medicine, City University of New York (CUNY), New York, NY 10031, USA; 4 Department of Medicine, Division of Hospital Medicine, Montefiore Medical Center, New York, NY 10461, USA

“In the midst of chaos, there is also opportunity”—Sun Tzu, The Art of War

The protean nature of the COVID-19 pandemic has necessitated unprecedented global coordination, cooperation, and ingenuity. Eleven variants of SARS CoV-2 and a multitude of subvariants have been identified [1], over half a billion humans have been infected, and more than 6 million people have perished [2]. Mirroring the meta-morphology of the virus, the medical and scientific community has proven to be extremely versatile, pushing the bounds of translational medicine further than ever before. Therapeutics, vaccines, and an explosion of data were generated, but perhaps the most impactful sequela has been learning how to produce meaningful research in shorter and shorter spans of time.

The pressing need for results, as well as inherent public and governmental pressure during a pandemic, stressed the integrity of the scientific process and required unconventional methods to work so many lines of inquiry in parallel. Finances and timelines factor heavily into therapeutic development, and repurposing existing medications for immediate use became an attractive option, as it is both cost-effective and time-saving [3]. Many clinicians will remember the state of COVID-19 research early on in the pandemic, which evolved from individual clinicians posting their experiences on Twitter, advancing to small, preliminary, and sometimes rushed clinical trials, and finally progressing to larger, more rigorous studies. Perhaps the largest and best tool utilized to answer these pressing clinical questions while striving to maintain intellectual fidelity was the adaptive platform trial [4]. Utilized in the RECOVERY [5], SOLIDARITY [6], and TOGETHER [7] trials (among others), this study type randomly assigns patients with a single disease to a group of carefully selected therapies of interest on the basis of a decision algorithm to determine whether they confer any significant benefit.

The RECOVERY trial is a seminal study establishing the benefit of dexamethasone, tocilizumab, and monoclonal antibody combination casirivimab/imdevimab, while finding no benefit in outcomes when administering aspirin, azithromycin, colchicine, convalescent plasma, lopinavir/ritonavir, and hydroxychloroquine. The study’s rapid design, enrollment, study size, and implementation are all testaments to a new phase in human research, marked by a globalist spirit and advanced logistical cooperation. However, this clinical trial is also a case study of the pitfalls of ‘stressed’ research. While perhaps unavoidable under the circumstances, the study used the same control group when comparing each of the intervention groups, the patients were not randomized within individual hospitals, and the data were unblinded to a data monitoring committee that performed five interim analyses.

In spite of the pressure brought to bear for immediate results, a large volume of literature was produced at the outset of the pandemic that withstood the test of time, was implemented in a timely manner, and potentially prevented a significant amount of morbidity and mortality. High-quality systematic reviews, observational studies, and meta-analyses were all utilized to great effect, resulting in protocols such as the one regarding steroid use implemented in our institutions near the advent of the pandemic [8], many of which were included in our very own special issue of COVID-19 Therapeutics. Additionally, open registries have proliferated with significant international buy-in, leading to collaboration that has turned COVID-19 research into a multidisciplinary venture [9,10,11]. These new, robust platforms have the profound potential to be applied broadly to emerging pathogens as well as other public health crises, creating an academic ecosystem where not only are data generated and shared more efficiently, but the collaborations to process and apply that information are already in place as well.

Public opinion has played an outsized role in shaping COVID-19 research and policy, as evidenced by the lopsided distribution of clinical studies dedicated to different therapeutics [12]. It is reasonable to attribute the heightened interest and investment into drugs such as hydroxychloroquine and ivermectin as a product of political and popular promotion generated by spurious results from early small and methodologically concerning studies [13,14]. Great care is required to strike a balance between heeding the needs of the general public whom we serve and shielding ourselves from the pressure they bring to bear.

An important question to ask is how our experiences have informed how we will approach the next pandemic. A vital first lesson is: do not rush. Fast science can be very bad science. We have created an unprecedented level of data sharing, and while the scale of the next pandemic may not be to the degree of COVID-19, the existing lines of communication should be maintained to more efficiently knit together small randomized-controlled trials, quality observational studies that utilize an array of statistical analyses in an effort to minimize the potential for confounding, or systematic reviews into more robust and higher-powered conclusions earlier on. The continued utilization of adaptable clinical trial models will certainly change the flexibility and scope of future inquiry.

The fruit of mankind’s collective scientific labors, however, is staggering. One modeling study projected that COVID-19 vaccination may have prevented 27 million SARS-CoV-2 infections, 1.6 million COVID-19–associated hospitalizations, and 235,000 COVID-19–associated deaths through September of 2021 [15] in the US; another study puts the number of deaths prevented worldwide at a whopping 19.8 million [16]. More than 5.33 billion people worldwide [17], nearly 70% of Earth’s inhabitants, are estimated to have been vaccinated to date, and despite the cycling of new, highly infectious variants, we have undoubtedly turned a corner in facing this pandemic and perhaps even closed a chapter. A generation of physicians has been tempered in the crucible of a pandemic and, in the process, have discovered the power of the individual to make a difference on a global scale. The imperative to investigate and research has been extended more solidly to the rank-and-file clinician through adaptive trial platforms, mRNA vaccines have proven their efficacy, and global registries have begun to change the way we approach data collection and processing. COVID-19 has proven to be a chameleonic adversary, but humanity has proven to be just as adaptable and, in the process, gained vital lessons to take into the future.
